# Behaviour of intrinsically disordered proteins in protein–protein complexes with an emphasis on fuzziness

**DOI:** 10.1007/s00018-017-2560-7

**Published:** 2017-06-08

**Authors:** Johan G. Olsen, Kaare Teilum, Birthe B. Kragelund

**Affiliations:** Structural Biology and NMR Laboratory (SBiNLab) and the Linderstrøm-Lang Centre for Protein Science, Department of Biology, University of Copenhagen, Ole Maaløes Vej 5, 2200 Copenhagen, Denmark

**Keywords:** IDP, Allovalency, Fuzzy complex, Signalling, Avidity, Disorder, Kinetics

## Abstract

Intrinsically disordered proteins (IDPs) do not, by themselves, fold into a compact globular structure. They are extremely dynamic and flexible, and are typically involved in signalling and transduction of information through binding to other macromolecules. The reason for their existence may lie in their malleability, which enables them to bind several different partners with high specificity. In addition, their interactions with other macromolecules can be regulated by a variable amount of chemically diverse post-translational modifications. Four kinetically and energetically different types of complexes between an IDP and another macromolecule are reviewed: (1) simple two-state binding involving a single binding site, (2) avidity, (3) allovalency and (4) fuzzy binding; the last three involving more than one site. Finally, a qualitative definition of fuzzy binding is suggested, examples are provided, and its distinction to allovalency and avidity is highlighted and discussed.

## Introduction

Signalling and regulation are essential to all living cells and are based on intermolecular interactions, most of which are mediated by proteins. A substantial fraction of proteins include large regions of disorder without clearly defined three-dimensional structure. Such intrinsically disordered proteins (IDPs) are not only very abundant—30–40% of all proteins in the human proteome are disordered or contain intrinsically disordered regions (IDRs) [1, 2]—they also constitute significant parts of membrane proteins [3, 4] and occupy pivotal positions in cellular regulation on all levels [5]. Some even display enzymatic activity [6]. Thus, IDPs are critically involved in key cellular processes and important for understanding life. Although IDP research has grown somewhat independent from traditional biology and biochemistry, it is conceptually important to follow the models, views and nomenclatures used generally for proteins, which have been developed over the past 120 years since Fisher proposed the lock-and-key model for ligand binding [7]. Thus, throughout this review the IDP is referred to as the ligand (L). The residues involved in binding are expected to be disordered, but that does not exclude the presence of ordered regions in other parts of the peptide chain. In the present discussion, ordered regions are assumed not to be involved in the interaction. The binding partner that may or may not be an IDP is referred to as the receptor (R), although this macromolecule does not need to be a receptor per se.

By definition, IDPs have high rotational freedom and sample a wide range of conformations [8–10]. Their hyper-dynamical nature renders them malleable and thereby potentiates their ability to bind multiple structurally diverse receptors, while retaining specificity. This conjecture implies that IDPs are superior to their folded counterparts when it comes to binding many different partners. Interestingly, the thermodynamics of the interaction between an IDP and a folded partner is essentially similar to the situation when two globular proteins interact, only compromised on average by around 2.5 kcal mol^−1^ due to loss of conformational entropy originating from the structuring of the disordered chain [11]. However, the distribution of states and the dynamics of the complexes vary. Some IDP binding sites become ordered upon binding to their receptor, a phenomenon called folding upon binding [12]. Several crystal and NMR structures of such complexes exist [13, 14] and they highlight details of the interactions [15–18]. In terms of kinetics, these are typical examples of simple two-state reactions, where the energy landscape of the complex is presented by a very deep well and one single structure can, in essence, represent the complex. At the other extreme, some ligands never ‘rest’ in complex with a receptor and there is no single conformation for the ‘bound-state’. In this case, the IDP ligand retains conformational freedom in the complex. Such interactions have recently been coined fuzzy complexes [19, 20] and a database has been established, collecting examples of the phenomenon [21]. Between these extremes, other binding modes are found. Earlier work has provided kinetic interpretations of those modes and their mechanisms of binding have been referred to as avidity and allovalency [22, 23]. In the following, we will describe the four different mechanisms in more details.

## Simple two-state binding

**Fig. 1:**
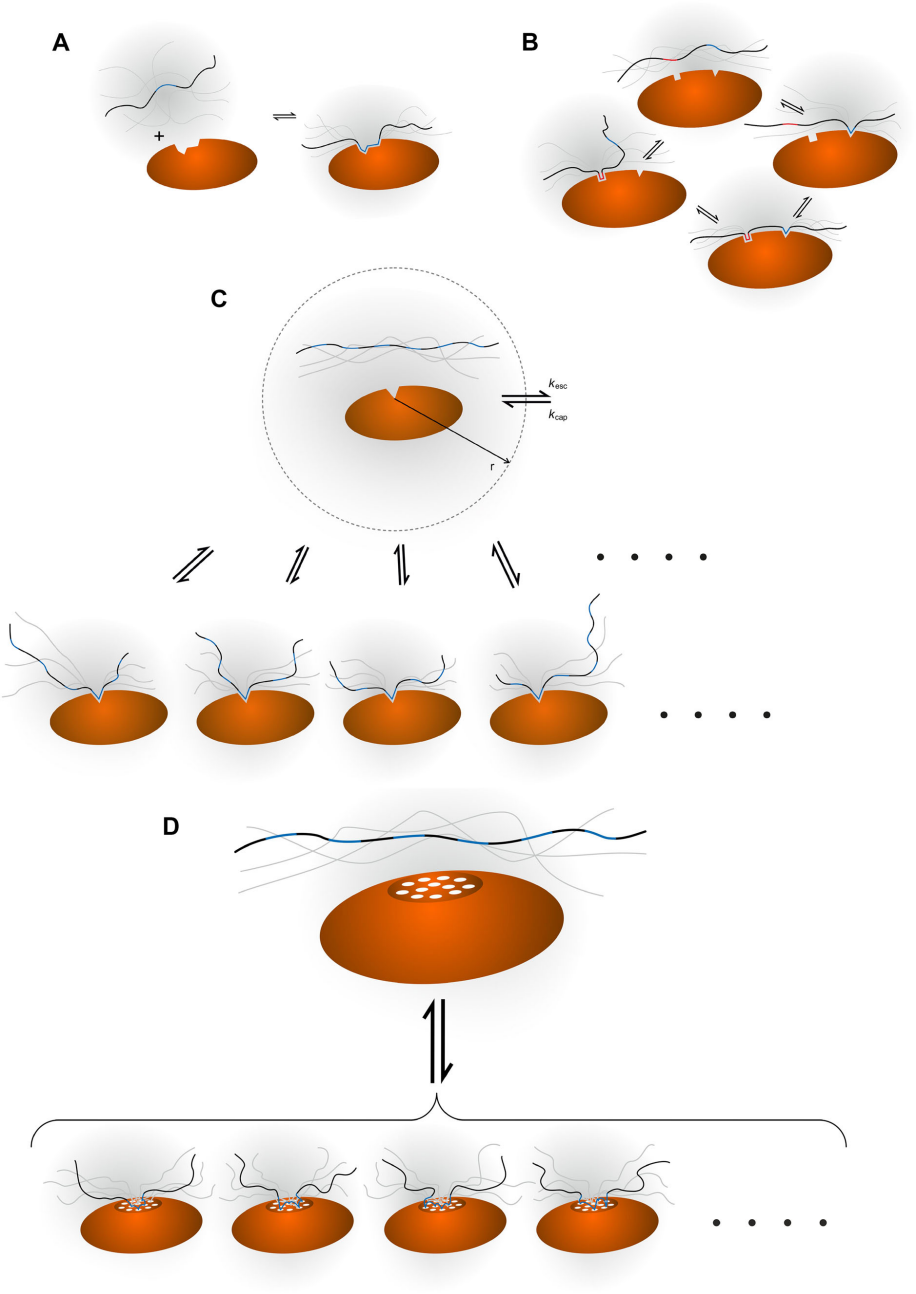
Illustration of four different binding mechanisms involving IDPs. Different ligand-receptor interactions involving an IDP ligand (*wavy black string*) and a macromolecular receptor (*orange oval*) are shown. The binding epitopes on the ligand are *highlighted in blue* or (*red*), whereas a binding site on the receptor is symbolised with an indentation. **a** Simple two-state binding, implying the existence of either the free or the bound state with no intermediates. There is a linear correlation between the concentration of ligand and the fraction of molecules in the bound state. **b** Avidity, where two or more epitopes on the ligand and a corresponding number of binding sites on the receptor will interact. If one interaction is established, a second binding event is more likely to occur due to the proximity of the additional interaction site(s). **c** Allovalency. A single binding site on the receptor can bind to several, identical epitopes along the ligand. When a bound ligand is released, the chance of rebinding is higher than anticipated from the ligand concentration alone, because of the very high epitope concentration close to the binding site on the receptor. An illustration of the capture sphere is shown with the corresponding rate constants. **d** Fuzzy complex. Both molecules have a number of interaction sites (shown as *white circles* on the receptor). An interaction site on the ligand is not restricted to connect with one specific interaction site on the receptor and vice versa. Thus, when one interaction is lost, the probability of forming another one because of the high local concentration of both ligand and receptor binding sites is much higher than what one would anticipate from ligand concentration alone. The smaller representation of the bound states in **c** and **d** is intentional, but not real, and is drawn for clarity only

The simplest description of the interaction between two molecules is that the molecules in their unbound state are separated from the complex state by a single transition state and that no intermediates are present (Fig. 1a). Such a scenario is often seen for the interaction between small molecules that exist mainly in one conformation. The interaction between complex macromolecules can often be approximated as two-state binding, even if the binding involves major conformational changes. The requirement for (approximate) two-state binding is that a single site of the ligand binds a single site on the receptor. In an ordered protein complex, that can be the end-result of a two-state reaction, each back-bone conformation adopts a narrow range of angular values in the bound state and all ligand atoms involved in binding are bound to specific receptor atoms, as crystal structures of such complexes show. Besides those cooperative events that occur within a single binding site between individual atoms, the binding energy is linearly dependent on the sum of interactions.

The binding is a second order reaction and there are no subsequent first order reactions. An example is the intrinsically disordered protein PUMA binding to the folded protein MCL-1 [24]. PUMA adopts an α-helix in the bound state and the helix forms in a coupled binding and folding event [24]. For the two-state reaction between a ligand (L) and its receptor (R) the following equilibrium exists:

1 $${\text{L}} + {\text{R}}\begin{array}{*{20}c} {k_{\text{on}} } \\ \rightleftarrows \\ {k_{\text{off}} } \\ \end{array} {\text{LR}}.$$

The binding constant given as the association constant, *K*_a_ is defined by the concentrations of the species in the solution at equilibrium:

2 $$K_{\text{a}} = \frac{{k_{\text{on}} }}{{k_{\text{off}} }} = \frac{{[{\text{LR}}]}}{{[{\text{L}}][{\text{R}}]}}.$$

## Avidity

Avidity was originally used to describe the binding between an antibody and an antigen, and is thus not exclusively an IDP phenomenon [25]. Avidity arises when two or more binding sites are present on the ligand, complementing two or more binding sites on the receptor (Fig. 1b). The binding sites on the ligand are connected by a linker and this linker ensures that once one site is bound to the receptor, other site(s) are spatially close to other receptor sites, resulting in cooperative binding, due principally to a lower entropic cost of binding more than one ligand [26]. Avidity requires the receptor and the ligand to have the same number of binding sites, where each site is unique and the sites cannot exchange. Once the ligand has bound one site, the probability of establishing an additional binding contact is much higher than for the first binding event and so forth, introducing cooperativity.

The first binding event is a second order reaction, whereas subsequent binding events are first order (pseudo-intramolecular) events. Thus, the entropic loss in subsequent binding events is lower.

If both of the two receptor-sites and the two ligand-sites are identical the order of binding is of no consequence. The first (second order) reaction is written as a simple two-state reaction:

3 $$\left( {\begin{array}{*{20}c} {{\text{L}} + {\text{R}}} \\ {{\text{L}}^{\prime } + {\text{R}}^{\prime } } \\ \end{array} } \right)\begin{array}{*{20}c} {k_{1} } \\ \rightleftarrows \\ {k_{ - 1} } \\ \end{array} \left( {\begin{array}{*{20}c} {\text{LR}} \\ {{\text{L}}^{\prime } + {\text{R}}^{\prime } } \\ \end{array} } \right),$$

and likewise, the second (first order) reaction is written:

4 $$\left( {\begin{array}{*{20}c} {\text{LR}} \\ {{\text{L}}^{\prime } + {\text{R}}^{\prime } } \\ \end{array} } \right)\begin{array}{*{20}c} {k_{2} } \\ \rightleftarrows \\ {k_{ - 2} } \\ \end{array} \left( {\begin{array}{*{20}c} {\text{LR}} \\ {{\text{L}}^{\prime } {\text{R}}^{\prime } } \\ \end{array} } \right),$$

where L′ and R′ refer to the sites involved in the second event. The first binding event can typically be studied experimentally using mutant proteins. The second step is trickier but the equilibrium constant for the second step, *K*_2_, may be estimated from the equilibrium constant for the first step, *K*_1_, by calculating the effective concentration of the second binding epitope in the vicinity of the receptor [25]:

5 $$\frac{{K_{2} }}{{K_{1} }} = \frac{V}{N}\frac{3}{{2\pi {\langle}r{\rangle}^{3} }},$$

where *V* is the volume of a sphere with *N* receptor binding sites, i.e. *V*/*N* is the concentration of receptor binding sites. ‹*r*› is the average distance between the two binding sites on the disordered ligand. If LR and L′R′ are identical, then *k*_off_ must be the same for both sites. This infers that the ratio between the on-rates, *k*_2_/*k*_1_, will be the same as that given for the equilibrium constants in Eq (5). Since the linker is disordered, its properties, in terms of dimensions, rigidity and modulation of dynamics and flexibility potentially by post-translational modifications, may influence the avidity by modulating ‹*r*› [27, 28]. Once the linker gets too long, the two sites decouple and the cooperativity is lost [25, 27].

## Allovalency

Allovalency is different than avidity and refers to the situation where more and identical receptor-binding sites (*n*) are positioned in tandem on an IDP, Fig. 1c. The concept was developed by Klein, Pawson and Tyers in 2003 [22] and discussed and elaborated by others [23, 29]. The identical binding sites on the ligand compete for a single binding site on the receptor and only one binding site on the ligand can bind at any given time [22], which is nicely exemplified by multiple phosphorylations on an IDP binding to and competing for the same site [30]. Although the affinity for one ligand is low, the presence and competition by multiple tandem sites increase the overall affinity. To explain this increased affinity a sphere centred at the receptor binding site was defined [22]. When a ligand molecule enters this sphere, e.g. is captured from the bulk with a rate *k*_cap_, it is entering the proximal region (P) from the free state (F), Fig. 1c. The ligand can then either escape again by diffusion to the free-state (L_F_) beyond the sphere with a rate of *k*_esc_, or bind with one of its binding sites to the receptor (the bound state, R·L) with a rate constant of *k*_on_. The reverse of this reaction happens with the rate constant *k*_off_



The values of *k*_esc_ and *k*_on_ depend on *n*, whereas *k*_off_ and *k*_cap_ do not, since at any given time, only one ligand site can occupy the binding site on the receptor, and entering the P zone from the F zone is a diffusion process that only depends on [L]. The rate constant *k*_esc_ thus decreases exponentially with *n*, introducing the cooperativity of the system.

7 $$k_{\text{esc}} (n) \approx \frac{{e^{{( - \frac{{k_{\text{on}} \left( n \right)\omega }}{35})}} }}{\omega },$$

where *ω* is the mean exit time of L from the proximal region P to bulk [22].

The defining example of allovalency is Sic1, an IDP from yeast, and its receptor Cdc4. The interaction depends on phosphorylation of up to ten serine and threonine residues on Sic1 [22]. Each of these phosphorylated epitopes can target a single binding pocket on Cdc4. The binding is cooperative, as when less than six sites are phosphorylated there is almost no binding. Phosphorylation of the sixth arbitrary group produces strong binding and further phosphorylation increases the affinity in a non-linear way. The fraction of bound Sic1 to Cdc4 is thus described as:

8 $$\frac{{[{\text{LR}}]}}{{(\left[ {\text{LR}} \right] + \left[ {\text{L}} \right])}} = \frac{1}{{1 + \frac{1}{{K_{\text{a}} [{\text{R}}_{\text{f}} ]}}}},$$

where [R_f_] is the concentration of the free receptor. The association constant, *K*_a_, becomes,

9 $$K_{a} = \left( {1 + \frac{{k_{\text{on}} }}{{k_{\text{off}} }}} \right)\frac{{k_{\text{cap}} }}{{k_{\text{esc}} }} .$$

Allovalent binding where ligand epitopes are created by post-translational modifications has the potential to function as a highly cooperative switch. Please note, that the allovalency model has been discussed and expanded beyond the present formulation by Locasale [29].

## Fuzzy binding

The term fuzzy complex was introduced by Tompa and Fuxreiter in 2008 [19] and the concept has been further refined and discussed, both by Fuxreiter and others [31–33]. The name is inspired by the mathematical term ‘fuzzy logic’ in which the true answer to a question can be no (0) or yes (1) or any value in between. Thus, analogously in a binding reaction, a ligand can be more than just fully bound to the receptor or completely free. As a further extension to this description, fuzzy complexes are ensembles of complexes, which are all needed to be able to fully describe the bound state (Fig. 1d).

In a wider perspective, all complexes at temperatures above 0 K are fuzzy. In one extreme, atomic vibrations cause fuzziness in a solid-state system. The opposite extreme can be illustrated by non-specific interactions between atoms or molecules in the gas state. In that light, treating fuzziness as a distinct biochemical phenomenon linked to IDPs seems artificial. Fuzzy complexes, however, challenge the view that a protein–ligand complex occupies a single structural state, a notion that is fuelled by the overwhelming amount of crystal structures of protein–ligand complexes deposited in the protein data bank. Obviously, X-ray crystallographic data are biased towards non-dynamic molecules. A fuzzy complex is dynamic in the bound state and occupies several conformational states. Consequently, crystallographic methods are not sufficient for realistic visualization. If a crystal could be grown with a fuzzy complex, the electron density at the binding interface would be the average of all the conformations present in the crystal and hardly possible to interpret. Alternatively, a single state is allowed in the crystal lattice, producing a misleading artefact, at best describing one out of many possible states. Having said that, it is important to mention an interesting study employing SAXS, NMR and X-ray crystallography to investigate the binding of an intrinsically disordered region of ribosomal S6 kinase1 (Rsk1) to its inhibitor S100B. The investigators caught different Rsk1 structures in different crystal forms of the complex and were able to describe the fuzzy complex using data obtained by all three techniques [34]. To our knowledge, the first fuzzy complexes discovered were the homodimerization of the intracellular region of the T cell receptor subunit *ζ* and, subsequently, the heterodimerization of the same receptor region with a folded protein (Nef protein core from simian immunodeficiency virus) [35]. Although dynamic dimers of IDPs exist [36], the existence of homo-dimers in the former publication has been challenged [37] and importantly, so has the initial notion that fuzzy complexes can form without any peak perturbations in the NMR spectra [38]. However, the nature of fuzzy complexes and the degree by which we currently understand them, combined with the degree by which their formation is manifested in changes in measureable parameters, challenge the current toolbox of structural biology. The development of new approaches, in which single molecules analyses are one important road ahead, is needed.

Fuxreiter et al. described fuzzy complexes as ‘protein complexes, where conformational heterogeneity of ID regions is retained and is required for function’ [32]. However, any bond between two functional groups will reduce the number of degrees of freedom of the system by thermodynamic definition. Assuming that conformational heterogeneity is proportional to the number of microstates of the system (definitions of conformational heterogeneity can be found here [39–41]), conformational heterogeneity cannot be completely retained, not even in a fuzzy complex, because each bound state has lower entropy than each unbound state.

Although the introduction of fuzziness and fuzzy complexes as concepts has been tremendously important for driving our understanding of IDPs, a stricter definition of fuzzy complexes is needed. Thus, to further advance the field, a formal definition of the fuzzy phenomenon in terms of molecular dynamics and kinetics is necessary. This definition must explain the affinity/kinetics and fuel the design of experiments that can directly test for the fuzzy phenomenon. Here we describe fuzziness as two or more ligand binding sites on the receptor being able to bind to two or more receptor binding sites on the ligand. In a sense this is a combination of two allovalency phenomena, one experienced by the ligand and one experienced by the receptor (Fig. 1d). We only describe this conceptually, and present no formalistic description, but refer to Vauquelin et al., who have described the simplest system formalistically where *n* = 2 for each partner of the complex [42, 43].

## What makes fuzzy binding special?

A fuzzy complex consists of an intrinsically disordered ligand and a receptor (which may and may not be disordered itself). The complex, once established, does not lead to a single ligand (and in some cases receptor) conformation, rather the ligand samples a large conformational space as functional groups bind and unbind the receptor. A functional group could be PO_4_^2−^, NH_3_^+^, O^−^, OH, CH_3_, a ring system etc., i.e. any functional group in a protein. The ligand-receptor sub-sites recombine during binding and individual interactions within the interface are short lived compared to the overall life-time of the complex. Furthermore, these individual interactions may recombine within the life-time of the complex. So one functional group on one of the proteins may be free to bind different functional groups on the other protein.

How is fuzziness different from the other mechanisms presented in this paper? Of the four modes of interaction described here, fuzziness is most easily confused with (or similar to) allovalency. The difference is that allovalency requires several identical binding sites on the ligand and a single ligand-binding site on the receptor [22]. In the case of allovalency, the cooperative dependency on the number of compatible sub-sites is reflected in *k*_esc_, because the probability that an unbound sub-site will rebind to the receptor, and thus prevent diffusion beyond the proximal region increases non-linearly with the number of sub-sites. However, whereas *k*_off_ is independent on the number of ligand sites in the case of allovalency, this will not be the case for fuzzy binding, if one accepts the definition above. In a fuzzy complex, both the ligand and the receptor contain several ‘sub-binding sites’ or compatible functional groups, and several of those groups can make contact simultaneously. This means that the observed *k*_off_ is dependent on the number of compatible functional groups and their individual *k*_off_s.

Although described individually, we anticipate the discovery of hybrid examples, where two or more receptor binding sites can bind several binding sites on the ligand and where both *k*_off_ and *k*_esc_ contribute to the cooperative effect. However, to be able to distinguish between the different mechanisms in a testable frame, we need formalistic descriptions. As far as we know, the exact formalistic definition of fuzzy complex formation in terms of how *k*_off_ depends on the number of groups has not yet been derived.

## Why fuzziness?

One might ask how fuzziness differs from other macromolecular complex formation processes. The difference between a fuzzy complex and unspecific contacts between macromolecules is that a fuzzy complex has a biological consequence. The affinity may be ‘high’ or ‘low’ but the important point is that the result of the interaction has biological outcome. A lower limit for apparent affinity is not possible to define and there is no reason to believe that a higher limit exists beyond which ordered structure is required. Some fuzzy complexes have reported *K*_d_ values in the nM range [34].

What can fuzziness offer biological systems that other kinds of complexes cannot? Perhaps the most obvious answer would be binding at low entropic cost, since a high degree of conformational heterogeneity is retained in the complex. However, in general, the negative (unfavourable) entropy change upon binding is more or less the same for IDPs as for folded proteins [11]. With the rather few protein complexes that have been classified as fuzzy so far, it remains to be seen if fuzzy complexes differ in this respect.

Since the cooperativity of fuzziness depends on *n*, one could imagine that in extreme cases this could lead to very strong binding, pushing the affinity into the pM range for large number of *n*. Since disorder is maintained in the complex, accessibility to modifying enzymes is not compromised. Thus, even though the affinity of the complex in its unmodified form is high, the lifetime of an individual conformational state is low, which allows for regulation *on the fly*. In this context, we notice that fuzzy complexes offer a scaffold for ideal rheostats [44], in which applying or removing post-translational modifications at the binding interface, can tune binding affinity.

In line with the notion that classical interactions between ordered macromolecules and interactions with IDPs in fuzzy complexes represent the extremes of a dynamics trajectory, fuzzy complexes may not require a fundamentally different explanation [38]. In the following, we provide two examples, which according to the definition highlighted above can be classified as fuzzy complexes.

### The complex between nucleoporin and nuclear transporter receptors

A protein that needs to enter the nucleus can do so by binding to a soluble protein called a nuclear transport receptor (NTR). In the nuclear pore these can bind to IDPs (or disordered regions in globular proteins) called FG-Nups (phenylalanine-glycine-rich nuclear pore proteins). The interaction between NTRs and FG-Nups were examined in vitro by single molecule FRET, NMR and molecular dynamics simulations [45, 46]. These studies showed that the IDP only undergoes subtle structural and dynamical changes in the complex. Each local interaction—the encounter between complementary functional groups—has low (mM) affinity but the apparent *K*_d_ is around 100 nM and *k*_*on*_ is remarkably high, approaching the theoretical diffusion limit (~10^9^ M^−1^ s^−1^). This hints that the interaction may not depend on the relative orientation of the molecules. The authors suggest that these unique kinetic characteristics make it possible to ‘grab’ the NTR proteins with high affinity, still unbind them efficiently and send them along to other Nups in the nuclear pore complex, until they have been transported through the pore with their passenger protein [45, 46]. Thus, these characteristics fulfil the expectations of a fuzzy complex.

### Clathrin heavy chain binding by AP180

The process of endocytosis involves dynamic interactions among molecules associated with the membrane. Molecular rearrangements result in invagination of the membrane and ultimately in a vesicle budding off. Central to this process is the association of protein AP180 with the N-terminal domain of clathrin heavy chain (TD). AP180 contains 12 degenerate motifs in the C-terminal 58 kD intrinsically disordered region, and each of these ~23 residues regions contain a DLL/DLF binding motif. Each TD domain has three AP180 receptor sites. Aspects of complex formation has been examined using NMR spectroscopy, analytical ultracentrifugation, isothermal titration calorimetry and X-ray crystallography [47–49]. NMR spectroscopy studies showed that the TD-bound and free state of an AP180 fragment containing two TD binding motifs retained disorder, but the spectra revealed chemical shift changes. The *K*_d_ values of the individual sites were determined to be around 200 µM. Interestingly, the *k*_off_ values were around 3000 s^−1^ and the *k*_on_ values were 1–2 × 10^7^ M^−1^ s^−1^, approaching those determined for the NTR—FG-Nup interactions described in the example above.

The two examples share very high *k*_on_ and *k*_off_ values. This may not be seen as a prerequisite for fuzzy complexes. A fuzzy complex can in principle exist in slow motion. In the case of allovalency, however, *k*_on_ must be higher than *k*_esc_. In spite of the resemblance between the two mechanisms, there are no constraints on *k*_on_ or *k*_off_ in fuzzy binding, except of course that *k*_on_/*k*_off_ > 1.

## Conclusion

Intrinsically disordered proteins form complexes with other proteins, and may do so by different binding mechanisms. Allovalency and avidity have been described formalistically in the literature whereas the phenomenon of fuzzy complexes has not. It has been put forward conceptually to describe a binding phenomenon associated with IDPs. In the present review, we argued that complete conformational heterogeneity cannot be retained in fuzzy complexes and that the cooperative dependence on the number of groups that can participate in binding (*n*) arises because both *k*_cap_ and *k*_off_ depend on the magnitude of *n*. Thus, an important conclusion is that IDP complexes with only one receptor-binding site are not strictly fuzzy, but must be described according to the formalisms of allovalency. Notably, both allovalency and fuzzy complexes are dependent on the ligand being an IDP, whereas two-state binding and avidity are not. To this end, the rationale for the existence of fuzzy complexes is discussed. Fuzzy complexes can have very low *K*_d_ values (nM or lower), but are not restricted to this, and the binding affinity has the potential to be rapidly regulated, for example by post-translational modifications, even when bound. This provides a versatility and swiftness in signal changes, and offers the possibility of rheostat regulation, which may not be possible in the interaction between folded proteins. Although we did not derive a formalistic description of fuzzy binding, we strongly encourage its derivation, which will allow for testable experiments to investigate fuzzy complex formation to the full.

## Glossary

Allovalency: A single receptor site binding to a ligand with several identical binding epitopes.
Avidity: Two or more receptor sites that bind to two or more ligand epitopes in a cooperative manner.
Fuzzy complex: An IDP (or IDR) binding to a receptor, constantly shifting the coupling of functional compatible groups, thereby retaining some of the conformational heterogeneity of the free molecule.
